# Insulin Resistance Is Associated with Prevalence of Physician-Diagnosed Urinary Incontinence in Postmenopausal Non-Diabetic Adult Women: Data from the Fourth Korea National Health and Nutrition Examination Survey

**DOI:** 10.1371/journal.pone.0141720

**Published:** 2015-11-03

**Authors:** Byung Il Yoon, Kyung-Do Han, Kyu Won Lee, Hyuk Sang Kwon, Sun Wook Kim, Dong Wan Sohn, Yong-Hyun Cho, U-Syn Ha

**Affiliations:** 1 Department of Urology, The Catholic Kwandong University of Korea, International St Mary’s hospital, Incheon, Korea; 2 Department of Biostatistics, College of Medicine, The Catholic University of Korea, Seoul, Korea; 3 Department of Urology, College of Medicine, The Catholic University of Korea, St Paul’s hospital, Seoul, Korea; 4 Department of Internal Medicine, College of Medicine, The Catholic University of Korea, Yeoido St Mary’s hospital, Seoul, Korea; 5 Department of Urology, College of Medicine, The Catholic University of Korea, Yeoido St Mary’s hospital, Seoul, Korea; The University of Tokyo, JAPAN

## Abstract

**Objective:**

To investigate the association between insulin resistance (IR) and urinary incontinence in Korean adult women by analyzing the data from the Korea National Health and Nutrition Examination Survey IV (KNHANES) 2007–2009

**Methods:**

A nationally representative sample of 5318 non-diabetic Korean women ≥19-years-of-age (3043 premenopausal and 2275 postmenopausal women) was included from KNHANES 2008–2010. IR was measured using the homeostasis model assessment of IR (HOMA-IR). Participants in the highest and lowest quartile of HOMA-IR were defined as insulin-resistant and insulin-sensitive respectively. Women who have current physician-diagnosed urinary incontinence were classified as having urinary incontinence.

**Results:**

Incontinence was found in 9.18% of the total population, 8.51% of the premenopausal population, and 10.86% of the postmenopausal population. The prevalence of incontinence increased with age, reaching a peak at 60-69-years-of-age. The prevalence of urinary incontinence increased significantly with higher HOMA-IR quartiles in pre- and post-menopausal women (p for linear association = 0.0458 and 0.0009 respectively). Among post-menopausal women, those in the highest quartile of HOMA-IR were significantly more likely to have urinary incontinence compared to those in the lowest quartile [adjusted odds ratio, 1.72; 95% confidence interval, 1.07–2.77]. However premenopausal population exhibited no association between incontinence and HOMA-IR quartiles

**Conclusion:**

Our results suggest that the prevalence of incontinence increased across HOMA-IR in non-diabetic adult women, and especially, IR might be a risk factor for incontinence in postmenopausal non-diabetic women.

## Introduction

Urinary incontinence is a major health issue for women, which diminishes their quality of life (QoL). In addition, this condition exacts emotional and hygienic impairments and economic expense [1,2].

Although several risk factors including body mass index (BMI), age, number of birth deliveries, and menopause have been suggested [3,4], further risk factors and the etiology of urinary incontinence remain unclear. Evidence is growing that diabetes is positively associated with urinary incontinence [5,6].

Metabolic disturbances including hyperglycemia, hyperinsulinemia, and insulin resistance (IR) are associated with the risk of lower urinary tract symptoms (LUTS) [7–9]. The pathogenesis of LUTS is considered to be a multifactorial process, and current concepts also include the theory of vascular impairment, with consecutive bladder hypoxia as a risk factor for LUTS [10]. Subsequent to IR, associated hyperinsulinemia leads to vascular endothelial dysfunction, abnormal lipid profile, and vascular inflammation, all of which promote the development of vascular impairment [11,12]. IR is regarded as the key feature leading to metabolic syndrome [13]. So, IR might promote LUTS through vascular impairment and might be a key step in early pathogenesis.

Research to date has focused on males. Data from females are scant. Moreover, the association between IR and urinary incontinence has not been investigated. We hypothesized that IR is associated with urinary incontinence. The aim of this study was to explore the association between IR and urinary incontinence among Korean adult women, using the homeostasis model assessment (HOMA) to assess IR.

## Methods

This study used the data from the Korea National Health and Nutrition Examination Survey IV (KNHANES) 2007–2009. KNHANES is a cross-sectional, nationally representative sample of the Korean non-institutionalized civilian population. and employs a rolling sampling design that uses a complex, stratified, multistage probability sample design. It constitutes a health interview, health examination, laboratory, and nutrition surveys. The study population for this analysis included adults ≥19-years-of-age who participated in all three parts of the survey. Subjects with diabetes mellitus or who were missing data on urinary incontinence or fasting plasma glucose or serum insulin were excluded. Participants who reported cessation of menstruation for more than 1 year formed the postmenopausal population. Women using hormone therapy, pregnant women, and women with premature menopause (before 40-years-of-age) or surgical menopause were excluded. The final study cohort included 5318 women (3043 premenopausal and 2275 postmenopausal). This study was approved by the Institutional Review Board of the Catholic University of Korea.

All general data including socioeconomic, lifestyle, and clinical variables have been fully described previously [14]. The definition of socioeconomic and lifestyle variables are as follows. Smoking status was categorized into two groups: Yes (those who were currently smoking and who had smoked 100 cigarettes or more in their lifetime) and No (non-smokers, and exsmokers, those who had smoked in the past but ceased smoking current smokers). Alcohol consumption status was defined as drinking more than once a month. Exercise was defined as strenuous physical activity performed for at least 20 min at a time at least three times a week, or mild physical activity performed for at least 30 min at a time at least five times a week. Highest diploma was divided into Yes and No by over high school education achieved. Household income was calculated as the family income, adjusting for the number of family members, and divided into Yes (below 4th quartile) and No. IR was calculated using (HOMA-IR with the following formula [15]: HOMA-IR (mmol/l × μU/ml) = [fasting glucose (mmol/l) x fasting insulin (μU/ml) [÷ 22.5. Individuals in the highest quartile of HOMA-IR were defined as insulin-resistant and those in the lowest quartile of HOMA-IR were defined as insulin-sensitive. The quartile value of HOMA-IR at 25^th^ percentile, 50^th^ percentile and 75^th^ percentile was 1.667, 2.076, and 2.657, respectively.

Individuals were classified as having urinary incontinence if they answered yes to the survey question "Do you have physician-diagnosed urinary incontinence?” Study participants were instructed that this question dealt with uncontrolled loss of urine in any situation accompanied by coughing, straining, sneezing, exercising, or feelings of urgency or pressure.

SAS version 9.2 (SAS Institute, Cary, NC, USA) was used for statistical analysis, using KNHANES sampling weights to acquire nationally representative estimates. The data in this study are presented as the mean ± SE or proportions (SE) for continuous or categorical variables respectively. The characteristics of subjects were compared according to menopausal status. Variables with skewed distributions were analyzed after logarithmic transformations. The prevalence of urinary incontinence was analyzed by general linear model, according to age and the HOMA-IR quartiles in subgroups: pre-menopausal and post-menopausal population. Multivariable logistic regression analyses were applied to examine the association between insulin resistance and urinary incontinence according to the HOMA-IR quartiles by menopausal status. The adjusted odds ratio (aOR) of urinary incontinence was calculated using the insulin-sensitive group as the reference. Calculations were made, adjusting for age, BMI, smoking, drinking, exercise, education, income, hypertension, and parity. A p-value <0.05 was considered statistically significant

## Results

### Prevalence of incontinence

**Fig 1:**
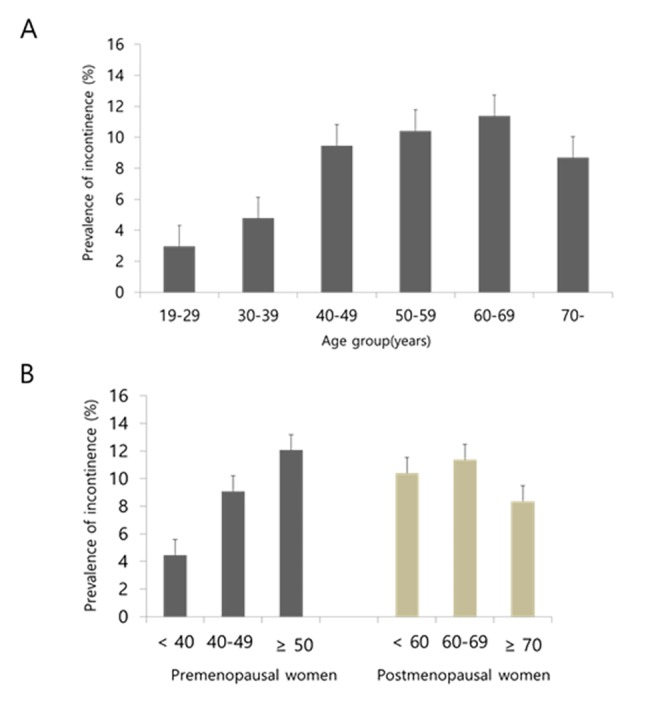
Prevalence of incontinence (A) according to age group, (B) according to age group by menopausal status. The error bars represent the upper standard error. (A) The prevalence of incontinence increased with age, reaching a peak at 60-69-years-of-age (p for linear association < 0.0001), (B) In the premenopause group, the prevalence of incontinence increased with age (p for linear association < 0.0001), in the postmenopause group, the prevalence of incontinence plateaued and was not appreciably different among each age group (p for linear association > 0.2008).

Incontinence was found in 9.18% of total population, 8.51% of the premenopausal population, and 10.86% of postmenopausal population. The prevalence of incontinence increased with age, reaching a peak at 60-69-years-of-age (p for linear association < 0.0001)(Fig 1A). In the premenopause group, the prevalence of incontinence increased with age (p for linear association < 0.0001), whereas the prevalence of incontinence plateaued and was not appreciably different among each age group after menopause (p for linear association > 0.2008) (Fig 1B).

### Comparison of clinical characteristics

Table 1 summarizes the general characteristics of the study population according to existence of incontinence, in the pre- and postmenopausal populations. In the premenopause group, significant differences were evident regarding exercise, education level, and parity. In the postmenopause group, only exercise was significantly different between population with or without incontinence. Table 2 presents the clinical characteristics of the study population according to existence of incontinence among the pre- and postmenopausal women. The mean BMI and waist circumference are significantly different between the incontinence and non-incontinence group In the pre- and postmenopausal women. Among the premenopausal women, mean age, triglyceride level, and the proportion of hypertension were significantly higher in those with incontinence. Among the postmenopausal women, levels of insulin and HOMA-IR were significantly higher in those with incontinence and mean concentration of high-density lipoprotein was lower in those with incontinence.

**Table 1:** General characteristics of the subjects according to menopausal status.

	Pre-menopausal women (n = 3043)			Post-menopausal women (n = 2275)		
	Incontinence			Incontinence		
	No (n = 2784)	Yes (n = 259)	P-value	No (n = 2028)	Yes (n = 247)	P-value
Smoking status	9.6(0.7)	7.9(2.6)	0.5407	8.7(0.7)	8.6(2.3)	0.9687
Alcohol consumption	49.2(1)	52.7(4.2)	0.655	25.3(1.2)	28.6(3.7)	0.3928
Exercise	25.5(1)	33.6(3.8)	0.0294	23.6(1.2)	33(3.5)	0.0041
Living in urban	84.6(1.9)	78.8(3.6)	0.0569	73.2(2.2)	71(4.4)	0.598
Highest diploma (over high school)	81.7(0.9)	69.7(3.7)	0.0002	22.4(1.4)	20.8(3.3)	0.6596
Household income (< 25%)	7.5(0.6)	8.6(1.8)	0.5349	32.9(1.4)	31.6(3.9)	0.7507
Living with spouse	90.8(0.6)	91(2.4)	0.964	64.5(1.3)	66.2(3.6)	0.6541
Parity*	3.0(0.0)	3.3(0.1)	0.0055	4.8(0.1)	4.8(0.2)	0.7533

Data are presented as Standard error.

*: presented as number (weighted%).

**Table 2:** Clinical characteristics of the subjects according to menopausal status.

	Pre-menopausal women			Post-menopausal women		
	Incontinence			Incontinence		
	No	Yes	P-value	No	Yes	P-value
Age	40.3±0.2	44.1±0.7	< .0001	62.8±0.3	62.1±0.8	0.3639
BMI(kg/m^2^)	23±0.1	24±0.3	0.0008	23.9±0.1	24.5±0.2	0.0048
WC(cm)	76.8±0.2	80.2±0.7	< .0001	81.4±0.3	84.4±0.6	< .0001
FPG (mg/dL)	91.1±0.2	92.2±0.7	0.1143	94.7±0.2	95±0.7	0.6691
Total cholesterol (mg/dL)	180.9±0.7	183.7±2.4	0.2662	202±1	202.8±2.4	0.7556
HDL-C(mg/dL)	55.5±0.3	53.9±1	0.1157	52.4±0.4	50.2±0.8	0.0155
LDL-C(mg/dL)	105.7±0.7	108±2.1	0.3005	123.5±0.9	125.4±2.2	0.4223
TGs*(mg/dL)	86.9(84.9,88.9)	99(91.1,107.6)	0.0053	119(116,122.1)	123.4(113.4,134.2)	0.3687
Insulin* (μIU/mL)	8.8(8.7,9)	9.2(8.7,9.8)	0.2767	9.1(8.9,9.3)	10.1(9.4,10.9)	0.0059
HOMA-IR*	2(2,2.1)	2.2(2,2.3)	0.1765	2.2(2.2,2.3)	2.5(2.3,2.7)	0.0095
Hypertension (%)	11.5(0.7)	19.6(3.4)	0.0044	44.2(1.3)	44.5(4.1)	0.9383

Data are presented as the means or % ± Standard error.

*: presented as geometric mean (95% confidence interval).

TGs and HOMA-IR were tested after logarithmic transformation.

BMI, body mass index; WC, waist circumference; FPG, fasting plasma glucose; HDL-C, high-density lipoprotein cholesterol; LDL-C, low-density lipoprotein cholesterol; TGs, triglycerides; HOMA-IR, homeostasis model assessment of insulin resistance

Definitions: Hypertension, systolic BP ≥130 mmHg or diastolic BP ≥85 mmHg or on antihypertensive medication.

### Prevalence of incontinence according to HOMA-IR quartiles

**Fig 2:**
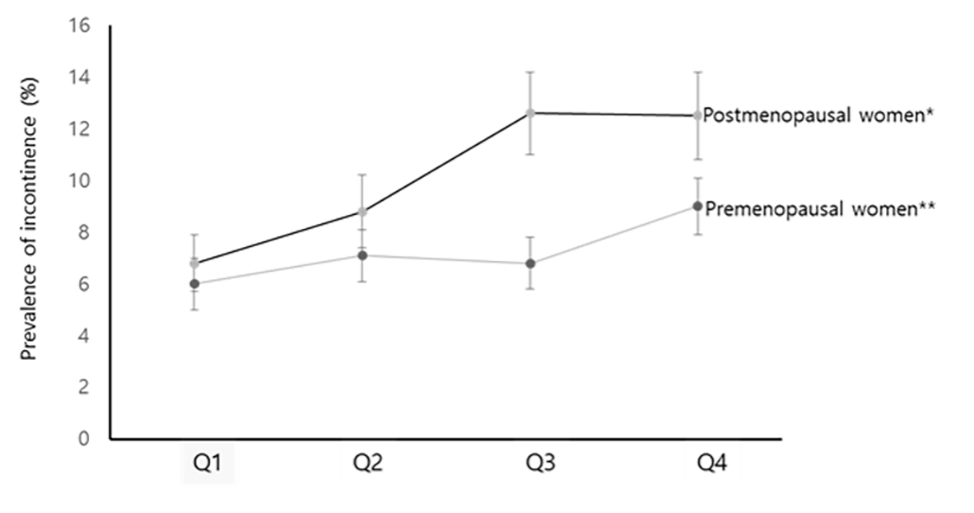
Prevalence of incontinence according to HOMA-IR quartiles. The error bars represent the standard error. The prevalence of incontinence increased across the HOMA-IR quartiles among premenopausal women and postmenopausal women. * Significant linear association from 1^st^ quartile to 4^th^ quartile (p for linear association = 0.0009). ** Significant linear association from 1st quartile to 4th quartile (p for linear association = 0.0458).

As expected, the prevalence of incontinence was higher in postmenopausal women. The prevalence of incontinence (presented as % ± standard error) was the lowest in 1^st^ quartile of HOMA-IR (6.0 ± 1.0 in premenopause, 6.8 ± 1.1 in postmenopause) and the highest in the 4^TH^ quartile of HOMA-IR (9.0 ± 1.1 in premenopause, 12.5 ± 1.7 in postmenopause) in both groups (Fig 2). A statistically significant trend of increasing prevalence of incontinence across the HOMA-IR quartiles was found among premenopausal women (p for linear association = 0.0458) and postmenopausal women (p for linear association = 0.0009). OR for incontinence increased significantly with the HOMA-IR quartiles in postmenopausal population, which means that post-menopausal women in the highest quartile of HOMA-IR were more likely to have incontinence than those in the lowest HOMA-IR quartile in an adjusted model for age, BMI, smoking, drinking, exercise, education, income, hypertension and parity, whereas premenopausal population exhibited no association between incontinence and HOMA-IR quartiles (Table 3).

**Table 3:** Adjusted ORs of incontinence according to HOMA-IR quartiles by menopausal status.

		Unweighted No. of urinary incontinence	Model1*	Model2^†^	Model3^‡^
Pre-menopausal Women	Q1	52	1	1	1
	Q2	58	1.2 (0.74,1.96)	1.17 (0.72,1.91)	1.12 (0.69,1.84)
	Q3	57	1.19 (0.75,1.88)	1.12 (0.71,1.78)	1.14 (0.72,1.80)
	Q4	80	1.57 (1.01,2.44)	1.37 (0.83,2.25)	1.48 (0.90,2.45)
	P		0.0412	0.2313	0.1156
Post-menopausal women	Q1	46	1	1	1
	Q2	56	1.33 (0.83,2.15)	1.29 (0.8,2.09)	1.33 (0.82,2.15)
	Q3	77	1.99 (1.27,3.12)	1.85 (1.17,2.92)	1.91 (1.21,3.04)
	Q4	85	1.99 (1.27,3.12)	1.75 (1.1,2.78)	1.72 (1.07,2.77)
	P		0.0007	0.0076	0.01

Data are presented as OR (95% confidence interval).

HOMA-IR, homeostasis model assessment of insulin resistance; OR, odds ratio; CI, confidence interval. Q1,2,3,4, HOMA-IR quartiles.

*adjusted for age.

†adjusted for age and BMI.

‡adjusted for age, BMI, smoking status, alcohol consumption, exercise, highest diploma, house income, hypertension and parity.

## Discussion

The main findings of this study are as follows: (1) the prevalence of incontinence increased across the HOMA-IR quartiles, regardless of the menopause state. (2) postmenopausal non-diabetic women in the higher quartile of HOMA-IR were more likely to be incontinent compared to counterparts in the lowest quartile independent of age, BMI, smoking, drinking, exercise, education, income, and hypertension. (3) elevation of IR is implicated as a risk factor of incontinence in postmenopausal non-diabetic women.

The underlying pathophysiology for these findings can be partly explained by the effect of IR on neurologic function, oxidative stress, and muscle mass and function IR may be important in the damage of neuronal function and pathogenesis of neurologic injury [16,17]. Insulin is a neurotrophic factor responsible for regulating neuronal growth, survival, and differentiation [16,18]. Considering the important neurotrophic role of insulin, disorder of insulin signaling during IR might result in neurodegeneration, although there are various theories concerning the pathogenesis of neuronal damage of IR. This neuronal damage is likely to lead to urethral sphincter dysfunction and altered bladder function. This explanation is supported by the report that insulin-resistant obese mice exhibit impairment of insulin action in the mucosal layer associated with overactive detrusor contraction and a reduction in bladder relaxant response to insulin, which induced incontinence [19].

Another possible explanation is that IR is closely related to increased oxidative stress due to mitochondrial dysfunction along with decreased mitochondrial size and mitochondrial DNA content [17,20]. IR can be accompanied by oxidative damage to tissue including bladder and urethra sphincter, which alters lower urinary tract function. Induction of oxidative stress followed by physiological alterations in the bladder has been described [21].

It is also possible that the loss of muscle mass due to IR might be independently associated with urinary incontinence. Recent compelling evidence concerning IR indicated that loss of skeletal muscle is an independent risk factor for the loss of skeletal muscle mass [22,23]. The pelvic floor muscles play an important role in the maintenance of continence. Pelvic floor muscle function is impaired in incontinent women [24,25], and pelvic floor muscle training is effective in reducing the symptoms of urinary incontinence [26]. The International Continence Society recommends pelvic floor muscle training as the first-choice treatment for stress and mixed urinary incontinence in women [27].

The distinctive feature of our study is that it is the first population-based study to suggest a significant association between IR and urinary incontinence in postmenopausal, non-diabetic Korean adults who participated in KNHANES 2008–2010. Although there is only one study regarding the association between diabetes and urinary incontinence, which study reported that diabetes is not independently associated with female incontinence [28], Actually diabetes and IR are clearly different disease, and we excluded participants with diabetes. Postmenopausal women in the higher quartile (Q3, Q4) of HOMA-IR were more likely to have urinary incontinence than those in the lowest quartile, independent of risk factors including age, BMI, smoking, drinking, exercise, education, income, and hypertension which are known risk factors. Our results could not show significant association in the premenopausal population (Table 3), although the prevalence of incontinence increased across the HOMA-IR quartiles among the premenopausal population (Fig 2). These results are noteworthy.

The other distinctive feature of the study is that analyses involved a nationally representative Korean population. The prevalence of urinary incontinence varies across racial and ethnic groups [29–31], Although the prevalence of urinary incontinence in KNHANES cannot be compared directly with that of NHANES in the United States due to difference in definitions of incontinence in the US, it has been observed that prevalence of incontinence in adults ≥20-years-of-age was 38.7% in NHANES [29] and peaked at 40-59-years-of-age. In Korea, the prevalence was lower (9.18%) than NHANES, reaching a peak at 60-69-years-of-age (Fig 1A). Our results are consistent with previous studies showing that the prevalence of incontinence increases with age [29,30,32]. We performed subgroup analysis dividing into pre- and postmenopausal populations; the prevalence was higher in postmenopausal women. However, the prevalence was not much different among each age group after menopause. On the other hand, significant trend of increasing risk across the HOMA-IR quartiles was found among postmenopausal women. This suggests the possibility that IR, rather than age, has more of an effect in the development of urinary incontinence in postmenopausal women.

Limitations of this study should be considered. One is the definition of urinary incontinence, which was from self-reported questionnaires; the definition to be used varied with each study. The NHANES definitions relied on an affirmative answer to the question “During the past 12 months, have you leaked or lost control of even a small amount of urine”, while for KNHANES incontinence was defined as a yes answer to the question to be uses is “Do you have physician-diagnosed urinary incontinence?” A considerable number of women with urinary incontinence would feel too shameful to go see a doctor and talk about their incontinence symptom. For these reason, the total burden of urinary incontinence in the study population could be substantially underestimated. This difference of definitions accounts for the higher prevalence in the US survey. But we enrolled participants with physician-diagnosed urinary incontinence to overcome the limitation from the quality of the answers, which may incur excessive impairment due to memorial and individual matters of the participants. The definition for urinary incontinence could vary according to survey. Urinary incontinence is divided into three major subtypes by the International Continence Society: stress urinary incontinence, urge urinary incontinence, and mixed urinary incontinence. We could not provide a subanalysis according to these subtypes, because subtype information is not available in KNHANES. In addition,

The other limitation is that KNHANES used the HOMA-IR index to evaluate IR, although the hyperinsulinemic–euglycemic glucose clamp, which can be prohibitively expensive and complex, is the gold standard for insulin sensitivity. However, HOMA-IR shows a high correlation with the hyperinsulinemic–euglycemic glucose clamp and can be reliably used in large-scale or epidemiological studies in which only a fasting blood sample is available to assess insulin sensitivity [33]. The bias due to HOMA-IR would not influence the outcome.

## Conclusions

The prevalence of incontinence increased across the HOMA-IR quartiles in non-diabetic adult women. Especially, Postmenopausal women in the higher quartile of HOMA-IR were more likely to have incontinence compared to counterparts in their lowest quartile independent of other risk factors. There may be a significant difference by menopausal status in the association between IR and urinary incontinence in non-diabetic adult women. So, elevation of IR might be a risk factor for incontinence in postmenopausal non-diabetic adult women.
